# Recent Progress in Research on Mitochondrion-Targeted Antifungal Drugs: a Review

**DOI:** 10.1128/aac.00003-23

**Published:** 2023-05-17

**Authors:** Yulin Qin, Jinxin Wang, Quanzhen Lv, Bing Han

**Affiliations:** a Department of Pharmacy, Minhang Hospital, Fudan University, Shanghai, China; b School of Pharmacy, Naval Medical University, Shanghai, People’s Republic of China

**Keywords:** antifungal agents, antifungal targets, mitochondria, electron transport chain

## Abstract

Fungal infections, which commonly occur in immunocompromised patients, can cause high morbidity and mortality. Antifungal agents act by disrupting the cell membrane, inhibiting nucleic acid synthesis and function, or inhibiting β-1,3-glucan synthase. Because the incidences of life-threatening fungal infections and antifungal drug resistance are continuously increasing, there is an urgent need for the development of new antifungal agents with novel mechanisms of action. Recent studies have focused on mitochondrial components as potential therapeutic drug targets, owing to their important roles in fungal viability and pathogenesis. In this review, we discuss novel antifungal drugs targeting mitochondrial components and highlight the unique fungal proteins involved in the electron transport chain, which is useful for investigating selective antifungal targets. Finally, we comprehensively summarize the efficacy and safety of lead compounds in clinical and preclinical development. Although fungus-specific proteins in the mitochondrion are involved in various processes, the majority of the antifungal agents target dysfunction of mitochondria, including mitochondrial respiration disturbance, increased intracellular ATP, reactive oxygen species generation, and others. Moreover, only a few drugs are under clinical trials, necessitating further exploration of possible targets and development of effective antifungal agents. The unique chemical structures and targets of these compounds will provide valuable hints for further exploiting new antifungals.

## INTRODUCTION

Fungal infections are a growing therapeutic concern among immunocompromised patients, as they are associated with high morbidity and mortality rates. Overall, approximately 1.7 billion people experience fungal infections and invasive fungal infections caused by *Candida*, Cryptococcus, and Aspergillus species, and these infections may lead to approximately 1.6 million deaths each year (1, 2). Five major classes of antifungal drugs are used for treatment in the clinical setting: polyenes, azoles, allylamines, pyrimidines, and echinocandins. Polyenes (e.g., amphotericin B), azoles (e.g., fluconazole, miconazole, itraconazole, and voriconazole), and allylamines (e.g., terbinafine) exert fungicidal and fungistatic effects by destroying the synthesis and function of ergosterol, a major component of the fungal cell membrane. Pyrimidines (e.g., flucytosine) act by disrupting DNA and RNA biosynthesis and interfering with nucleic acid synthesis. Echinocandins inhibit β-1,3-glucan synthase and subsequently disrupt fungal cell wall organization. Although existing antifungals are widely applied in the clinical setting, they still have many limitations, such as toxic side effects, narrow-range antifungal spectra, and antifungal resistance. Long-term regimen courses and overuse of azoles, polyene, and echinomycin have led to the high prevalence of drug resistance (3). According to the Centers for Disease Control and Prevention, approximately 7% of *Candida* isolates causing bloodstream infection are resistant to fluconazole, and nearly 4.9% of patients with severe disease died of drug-resistant *Candida* spp. infection (4). It is estimated that more than 60% of patients with candidemia receive echinocandin therapy, and 8.0% to 9.3% of Candida glabrata bloodstream isolates showed resistance to echinocandin, which has been a major obstacle for treatment (5). New-generation triazoles, such as posaconazole and voriconazole, have the advantages of extended-spectrum activity and availability for both oral and intravenous medication; however, significant drug-drug interactions, adverse effects, and cross-resistance limit their clinical applicability (6). To solve these problems, it is necessary to put in devoted efforts for developing new drugs. We present a review of novel antifungal drugs targeting mitochondria, which in turn provides potential strategies and targets for antifungal development.

## ADVANTAGES OF MITOCHONDRIA AS TARGETS OF ANTIFUNGAL DRUGS

The mitochondrion is essential for fungal cell survival and is a pivotal factor for the fungus to acquire adaptive abilities, including morphogenesis, virulence, and drug resistance (7). Mitochondria can be explored as novel targets for antifungal leads owing to their important role in fungal pathogenicity (8). More than 1,000 proteins are present in the mitochondria, most of which are encoded by the cell nucleus and highly conserved among species (9). Thus, exploring new mitochondria-targeted antifungal agents is challenging because of the close evolutionary relationship between fungi and humans. Moreover, fungal mitochondrial functions, including cellular respiration, oxidative phosphorylation, ATP synthesis, genetics, metabolic transport, drug resistance, and others, are highly conserved among different fungal species. The realization of diverse mitochondrial functions depends on the action of functional proteins (10). Some mitochondrial proteins and genes are species specific, and extra attention should be paid for the discovery of selective antifungal agents. Fungus-specific proteins are involved in the following mitochondrial processes: electron transport chain (ETC), carbon source utilization, protein sorting and trafficking, stress response reactions, and mitochondrial dynamics (8). However, existing studies on mitochondria-targeted antifungal drugs are limited and have mainly focused on ETC complexes (complexes I, III, and IV), which catalyze enzymes (e.g., dihydroorotate dehydrogenase [DHODH]) and mitochondrial phosphate trafficking (e.g., Mir1) (Table 1). A large proportion of potential compounds cause only mitochondrial dysfunction, which includes mitochondrial respiration disturbance, increased intracellular ATP, reactive oxygen species (ROS) generation, and other processes, but there is a lack of studies on other processes as targets (Table 1).

**TABLE 1:** Summary of antifungal agents recently explored that target mitochondria

Agent	Stage	Target	Antifungal spectrum	*In vitro* MIC (μg/mL)	*In vivo* efficacy^*a*^	Cytotoxicity IC_50_ (μg/mL)^*b*^	Selectivity (fold)^*b*^	Reference(s)
T-2307	Phase II clinical trials	CIII, CIV	*Candida*	0.00025–>4	Murine models of invasive candidiasis, aspergillosis, and cryptococcosis	N/M^*c*^	>1,000	28–31, 35, 39
			Aspergillus	0.125–4				
			Cryptococcus	0.0039–0.06				
F90138	Phase IIb clinical trials	DHODH	Aspergillus, *Coccidioides*	≤0.06	Murine models of invasive aspergillosis and coccidioidomycosis	N/M	>2,200	40, 42, 43
Ilicicolin H	Preclinical	Cytochrome *bc*_1_ reductase (CIII)	*Candida*	0.01–5.0	Murine models of invasive candidiasis and cryptococcosis	N/M	>1,000	44, 46
			Cryptococcus	0.1–1.56				
			Aspergillus	0.08				
Inz-1	Preclinical	Cytochrome *bc*_1_ (CIII)	C. albicans	0.44 (MIC_50_)	N/M	8.52 (HepG2)	>19.0 (HepG2)	48
Inz-5	Preclinical	Cytochrome *bc*_1_ (CIII)	C. albicans	0.15 (MIC_50_)	Murine models of invasive C. albicans infection (combined with FLC^*d*^)	4.13 (HepG2)	27.8 (HepG2)	48
ML316	Preclinical	Mir1	*Candida*	0.008–1.156	Murine model of oropharyngeal candidiasis	>1.0 (HepG2)	>125 (HepG2)	7
19ak	Preclinical	CI	*Candida*	0.125–1	G. mellonella model of C. albicans infection	26.79 (HK2)	214.32 (HK2)	49 – 51
			Cryptococcus	0.25–0.5		6.972 (16HBE)	55.78 (16HBE)	
			Aspergillus	0.5		10.3 (HepG2)	82.4 (HepG2)	
SM21	Preclinical	Mitochondrial function	*Candida*	0.2–1.6	Murine model of invasive C. albicans infection	3.54 (HOK)	17 (HOK)	52 – 54
Berberine	Preclinical	Mitochondrial function and Mdr1	*Candida*	4–16	Murine model of invasive FLC-resistant C. albicans infection	>90.02 (HepG2)	>5.62 (HepG2)	57, 59
			Cryptococcus	2–16				
			Aspergillus	4				
Resveratrol	Preclinical	Mitochondrial function	*Candida*	20–>300	N/M	46 μg/mL (HCT-116)	<2.3 (HCT-116)	60, 61, 66
			*Trichophyton*	25–50				
			*Epidermophyton floccosum*	25–50				
			*Microsporum gypseum*	25–50				
Chiloscy-phenol A	Preclinical	Mitochondrial function	*Candida*	8–32	Caenorhabditis elegans model of C. albicans infection	11.48–14.08 (HBE)	0.40–1.60 (HBE)	67
Floricolin C	Preclinical	Mitochondrial function	C. albicans	8–16	Caenorhabditis elegans model of C. albicans infection	N/M	N/M	68, 69
Kalopanax-saponin A	Preclinical	Mitochondrial function, cell membrane	*Candida*	8–16	C. elegans model of C. albicans infection	N/M	N/M	70, 71
Mefloquine derivatives	Preclinical	Mitochondrial function, vacuole	*Candida*	2–8	N/M	2.14–2.88 (HepG2); 4.47–6.02 (A549)	0.27–2.88 (HepG2); 0.56–6.02 (A549)	72
			Cryptococcus	1–4				
			Aspergillus	2–8				
*Carica papaya* L. seed extract	Preclinical	Mitochondrial function	*Candida*	4–16	N/M	N/M	N/M	73, 74
Rosmarinic acid	Preclinical	Mitochondrial function	*Candida*	0.1–0.2	N/M	360.31 (HepG2)	1,801.55–3,603.1	75, 76
P-113Du and P-113Tri	Preclinical	CI, NADH dehydrogenase	C. albicans	0.78–1.56	N/M	>400 (S-G)	>256 (S-G)	11, 80
ToAP2D Peptide	Preclinical	Mitochondrial function, cell apoptosis and necrosis	*Sporothrix globose*	156	Murine footpad model of *S. globosa* infection	N/M	N/M	81, 82

a Based on increased survival or reduced CFU in fungal infection models.

b Cell lines: HepG2, hepatoma cells; HK2, human kidney-2 cells; HBE, human bronchial epithelial cells; HOK, human oral keratinocytes; HEK293, human embryonic kidney cells; S-G, Smulow-Glickman human gingival epithelial cells; HCT-116, human colorectal carcinoma cells; A549, human epithelial cells.

c N/M, not mentioned.

d FLC, fluconazole.

The mitochondrion is the powerhouse of cell metabolism and produces energy through oxidative phosphorylation (OXPHOS), which involves the ETC (11). The fungal ETC includes five respiratory complexes (complexes I to V [CI to CV]), of which NADH-ubiquinone oxidoreductase (CI), ubiquinol-cytochrome *c* oxidoreductase (CIII), and cytochrome *c* oxidase (CIV) are responsible for generating proton gradients. Once the proton gradients are established, the F_1_F_0_ ATP synthase (CV) can produce ATP by binding free ADP to free phosphate. Seven CTG clade-specific mitochondrial subunit proteins on the ETC are required for the expression of various ETC complexes and respiratory function and may be potential antifungal drug targets (12). Among the CTG clade-specific mitochondrial subunits, Nue1, Nue2, Nuo3, and Nuo4 are subunits of CI. Qec1 is a subunit of CIII. Coe1 and Coe2 are subunits of CIV (13). Mitochondrial CI is the largest and most complicated enzyme complex in OXPHOS and is responsible for pumping electrons into the ETC and generating intracellular ROS. Deficiency of CI will result in cellular ROS accumulation, apoptosis, and increased susceptibility to fluconazole in Candida albicans (14–16). Among these CI subunits, three proteins (Nuo1, Nuo2, and Ndh51) contribute initially to mitochondrial respiration and then to cell wall integrity, which is highly related to the susceptibility of antifungal agents. Moreover, Nuo1 and Nuo2 are fungus-specific CI subunits, which may be more suitable for antifungal targets (8, 16). Goa1, a CI regulatory protein, merely exists in the CTG clade of *Candida* spp., and it plays a major role in membrane transportation, intracellular ROS regulation, and trafficking between peroxisomes and mitochondria (17, 18). Nuo1 and Nuo2 are fungus specific, and *NUO1*/*NUO2* deficiency will cause CI disassembly, reduced oxygen consumption, increased intracellular ROS, and decreased chronological aging *in vitro* and decreased virulence in a murine model (19). Ndh51 is broadly conserved among species and is responsible for NADH binding and oxidation. *NDH51* deletion causes suppressive cAMP- and protein kinase A-mediated ergosterol synthesis and results in an ≈47% decrease in ergosterol levels and fluconazole hypersensitivity of C. albicans (20). Unlike other complexes, CII is not involved in the formation of a proton gradient. CIII is a cytochrome *bc*_1_ complex that mediates the transfer of electrons from ubiquinol to cytochrome *c* (21). CIV (cytochrome *c* oxidase) acts as an electron acceptor in the reduction reaction of O_2_ to H_2_O. CIII and CIV are involved in the regulation of antifungal susceptibility, as mutants of these two subunits show hypersusceptibility to fluconazole (12).

## APPROACHES TO DELIVER MEDICAL AGENTS TO MITOCHONDRIA

The mitochondrion is considered a therapeutic target for a variety of diseases (cancer, cardiovascular diseases, neurological diseases, and infections), not only because of its important functions in cell viability but also because of its mitochondrial targeting characteristics (22). Several effective strategies for mitochondrial targeting have already been developed (23, 24). Pharmacophore molecules linked to lipophilic cation moieties, such as triphenylphosphonium (TPP^+^), can successfully penetrate biological membranes and realize the negatively charged intramitochondrial localization (23). Thus, TPP^+^ has been widely used in the delivery of antineoplastic drugs and diagnosis, including conventional chemotherapeutic drugs, photosensitizers for photodynamic and photothermal treatment and a combination of them (24). Chang et al. demonstrated that TPP^+^ confers protection to candidate compounds from being expelled by efflux pumps and helps the compounds to exert fungicidal effects by aiding them to localize within the mitochondrial matrix (25). Moreover, targeting peptides such as Szeto-Schiller peptide and mitochondria-penetrating peptide facilitate peptide-tagged target compounds to localize within the mitochondria (26, 27). Additionally, rhodamine, cyanine, and pyridinium ions are small molecules that exhibit mitochondrial-targeting properties (24).

## MITOCHONDRIA-TARGETED ANTIFUNGALS WITH SPECIFIC TARGETS

### Arylamidine T-2307.

T-2307 has shown broad-spectrum antifungal activity against fungal pathogens, including *Candida*, Aspergillus, and Cryptococcus spp. (28–31). As demonstrated through *in vitro* testing, the MIC of T-2307 ranges from 0.00025 to 0.0039 μg/mL against *Candida* species, which is significantly lower than its minimum fungicidal concentration (MFC), indicating that T-2307 has fungistatic effects against *Candida*. In contrast, T-2307 has shown fungicidal activity against Aspergillus nidulans and Aspergillus niger in the MFC range of 0.0313 to 0.0625 μg/mL and fungistatic activity against other Aspergillus spp. (29). T-2307 also inhibits Cryptococcus neoformans and Cryptococcus gattii in the MIC range of 0.0039 to 0.06 μg/mL. Moreover, T-2307 is effective against drug-resistant fungal isolates both *in vitro* and *in vivo*, which highlights its potent antifungal activities against echinocandin-resistant C. albicans and Candida glabrata and azole-resistant C. albicans (29, 32–34). T-2307 has also demonstrated antifungal effects on Candida auris infection, which is refractory for its resistance to several antifungal agents (30).

T-2307, an aromatic diamidine, also includes pentamidine and furamidine, two widely used medicines for pneumocystosis and trypanosomiasis treatment (35). As pentamidine and furamidine cause collapse of mitochondrial membrane potential (MMP) in yeast, T-2307 is speculated to demonstrate the same effects (36). Several studies have unraveled the mechanism of action of T-2307 and revealed that it is an inhibitor of mitochondrial respiratory chain CIII and CIV. T-2307 causes the disruption of MMP and mitochondrial dysfunction (35). However, the required concentration of T2307 to induce the collapse of the fungal MMP is significantly higher than its MICs (37). Further research revealed that T2307 can be transported into C. albicans cells through an Agp2-regulated high-affinity spermine and spermidine carrier (38). T-2307 has high selectivity for fungal mitochondria over mammalian cells, which makes it a potent drug candidate for fungal infection (34, 39). T-2307 is currently undergoing phase II development as ATI-2307. According to the results of current clinical research, ATI-2307’s potential target of indications will include cryptococcal meningitis and invasive candidiasis.

### F90138.

F90138 (also named olorofim) is a novel antifungal agent categorized under orotomides, and it was first identified by F2G Ltd. during screening of a small-molecule library against Aspergillus fumigatus. This compound is highly effective against most Aspergillus species, with a MIC of <0.1 μg/mL (40). However, F90138 is inactive against yeasts and *Mucorales* (41). F90138 targets DHODH, an essential mitochondrial enzyme that catalyzes the conversion of dihydroorotate to orotate in the *de novo* pyrimidine biosynthesis pathway (40). The selectivity of F90138 is more than 2,200-fold higher A. fumigatus DHODH than against the mammalian enzyme, which indicates that F90138 is a potential inhibitor that binds to fungal DHODH. *In vivo* experiments have shown that F90138 treatment improves survival and reduces fungal burden in murine models of neutropenia and chronic granulomatous disease with invasive aspergillosis (42). Similar results have been observed in a murine model of central nervous system coccidioidomycosis (43). F90138 is currently in development in a phase IIb clinical trial for the treatment of invasive fungal infections, including *Lomentospora prolificans*, *Scedosporium* spp., Aspergillus spp., and other drug-resistant fungi infections in patients who have limited treatment options (https://clinicaltrials.gov/ct2/show/NCT03583164).

### Ilicicolin H.

Ilicicolin H is a potent and broad-spectrum antifungal agent that has shown inhibition of *Candida*, Cryptococcus, and Aspergillus spp., with MICs ranging from 0.04 to 1.56 μg/mL (44). It is a natural polyketide synthesized by nonribosomal peptide synthase isolated from the fungus *Gliocladium roseum* and discovered by screening natural product extracts against C. albicans (45). Ilicicolin H exhibits inhibition by targeting cytochrome *bc*_1_ reductase (mitochondrial respiratory CIII) (8, 46). However, ilicicolin H displays strong binding potency with plasma protein, resulting in increased MIC values and decreased antifungal activity. The biological activity of this compound can be improved by chemical structure modification and biotransformation, generating 4,19-diacetate and 19-cyclopropyl, respectively, with considerable antifungal activities and selectivities. The plasma protein binding of 4,19-diacetate and 19-cyclopropyl acetate was reduced by approximately 20-fold (46). Biosynthesis also largely contributes to the production of ilicicolin H analogs, which will help solve the high plasma binding problem. Ilicicolin J is an analog of ilicicolin H produced by heterologous expression of ilicicolin H and displays similar antifungal activity as that of ilicicolin H (47). Further studies on the structure-activity relationships and structural modification of ilicicolin H derivatives are needed.

### Inz-1 and Inz-5.

Inz-1 is an indazole compound obtained during a phenotypic high-throughput screening with an aim to search for potent antifungal agents against azole-resistant strains (48). The high fungal selectivity (19-fold over that of humans) and growth inhibition (50% inhibitory concentration [IC_50_] of 1.6 μM) combined with chemical tractability of Inz-1 have made Inz-1 a potential lead compound for further research and development. Inz-1 is a cytochrome *bc*_1_ inhibitor that inhibits the mitochondrial inspiration of C. albicans and Saccharomyces cerevisiae, thus potently inhibiting their growth in a dose-dependent manner. However, the poor blood stability (<1% remaining after 1-h incubation) and microsome stability of Inz-1 (<1% remaining after 15-min incubation) limit its application. A series of analogs of Inz-1 were synthesized and evaluated to identify a superior inhibitor of fungal cytochrome *bc*_1_. Among the synthesized compounds, Inz-5 was found to be the optimal compound, with significantly enhanced potency (IC_50_, 0.381 μM), fungal selectivity (27.8-fold over that of humans), and modestly improved microsomal stability (19.5% remaining after 15-min incubation), although further optimization is still required. Moreover, Inz-5 has proven to significantly prevent the emergence of azole resistance and exert fungicidal effects combined with fluconazole in a murine model. Furthermore, Inz-5 substantially exhibits greater inhibition (28-fold) of the cytochrome *bc*_1_ activity in C. albicans compared to Inz-1. The disabled cytochrome *bc*_1_ creates an obstacle for fungal nonfermentation carbon source utilization and hyphal growth and is easily attacked by macrophages. Although further modifications are still needed, indazole antifungal agents (Inz-1 and Inz-5) have uncovered a new therapeutic strategy for resolving fungal drug resistance by inhibiting mitochondrial respiration.

### ML316.

ML316 is a novel selective inhibitor of the fungal mitochondrial phosphate carrier Mir1, and it exhibits potent antifungal effects, especially on azole-resistant C. albicans (7). Antifungal activity studies have indicated that ML316 exerts fungicidal activity against fluconazole-sensitive C. albicans strains with an MIC range of 0.008 to 0.063 μg/mL and against azole-resistant C. albicans strains with an MIC range of 0.05 to 0.5 μg/mL, which is significantly superior to that of fluconazole (7). As for cytotoxicity testing, ML316 has no effects on the viability of 293T and HepG2 cell lines. In a mouse model of oropharyngeal candidiasis, ML316 alone markedly reduced the fungal burden by 100-fold and caused a greater reduction by >1,000-fold when combined with fluconazole, indicating the role of ML316 in enhancing azole activity. However, studies on ML316 pharmacokinetics have revealed its poor plasma stability, which results in rapid clearance and a short half-life. The proven target of ML316, Mir1 is located in the inner mitochondrial membrane and acts as a shuttle for transporting inorganic phosphate from the cytoplasm into the inner matrix of fungal mitochondria to participate in ATP synthesis. Thus, ML316 exerts antifungal effects by inhibiting the activity of Mir1, transportation of inorganic phosphate, and disruption of ATP production and fungal respiration. Therefore, targeting Mir1 provides a novel and much-needed therapeutic strategy for the growing trend of drug-resistant fungal infections.

## MITOCHONDRIA-RELATED ANTIFUNGAL HITS

### 19ak.

As a thiosemicarbazone derivative, 19ak is a potent agent for fungal infection treatment, as it acts by inhibiting mitochondrial respiration by retarding mitochondrial respiratory chain CI activity (49). 19ak exhibits potent antifungal activity against C. albicans (MIC, 0.125 μg/mL), C. neoformans (MIC, 0.5 μg/mL), and A. fumigatus (MIC, 0.5 μg/mL). The MFC value for 19ak was 64 μg/mL. *In vivo* experiments conducted in Galleria mellonella infection model have shown that 19ak exerted considerable therapeutic antifungal efficacy with fluconazole. 19ak also has low cytotoxicity (IC_50_, 26.79 mg/mL in the HK2 cell line) and a high selectivity index (214.32), indicating its potential for further study. Although the mechanism of action of 19ak has been related to the inhibition of CI and iron chelation, the exact target remains unclear (50, 51). As thiosemicarbazones are compounds with antifungal potency and high selectivity, understanding their mechanism of action and pharmacokinetics provides more hints for exploring new antifungals.

### SM21.

A high-throughput screening of a library containing 50,240 small molecules showed that SM21 is a potent inhibitor of the yeast-hypha transition (52). Further evaluation of its *in vitro* fungistatic activity has revealed that SM21 is effective against a range of *Candida* spp., with MICs ranging from 0.2 to 1.6 μg/mL, and it has strong antibiofilm properties, with 50% viability reduction. SM21 is active against fungal drug-resistant isolates of *Candida* (MICs, 0.5 to 1.0 μg/mL). SM21 significantly improved the murine survival rate in an invasive candidiasis model by 100% in 5 days. The fungal burden and lesions are reduced in kidney and oral mouse models after treatment with SM21. A cytotoxicity assay of SM21 with human oral keratinocytes revealed a selection index of 17.0 for this compound, which indicated that SM21 is a drug candidate that does not cause significant toxicity to human cells (53). SM21 has effects on the dysfunction of mitochondrial components, including collapsed MMPs, reduced ATP generation, increased ROS production, and decreased antioxidant potency (54). However, the exact target of this novel antifungal molecule remains unknown.

## NATURAL ANTIFUNGAL HITS AFFECTING MITOCHONDRIAL FUNCTION

Natural products are an immense treasure for identifying new bioactive molecules, including some important antifungal agents. A wide variety of antifungal drugs have been obtained by isolating them from natural products (amphotericin B, nystatin, natamycin, and pentamycin) or by modifying natural products (anidulafungin, caspofungin, and micafungin) (55). However, no mitochondrial-targeting antifungal agents have been approved for marketing.

In recent years, some progress has been made in developing mitochondria-targeting antifungal compounds from natural products. Berberine is a common isoquinoline alkaloid with extensive pharmacological activities, including anti-inflammatory, anticancer, and antimicrobial activities, and is effective for *Candida* infection treatment, especially for fluconazole-resistant *Candida* isolates (56, 57). Berberine has shown a synergistic antifungal effect with azoles, especially with fluconazole (58). It also has excellent *in vivo* antifungal activity, wherein it significantly increases the mean survival time of mice infected by *MDR1*-overexpressed strains and displays selective fungal cytotoxicity on mouse cells compared with that on human cell lines. Berberine hijacks the multidrug efflux pump Mdr1, leading to berberine entry and accumulation; subsequently, berberine targets the mitochondria for impairing mitochondrial CI and interrupting ETC, finally resulting in the killing of Mdr1-overexpressing C. albicans cells (59). Thus, the potential clinical application of berberine in overcoming drug resistance is noteworthy.

Similarly, resveratrol, a natural polyphenolic compound, exhibits a wide range of antimicrobial activities on pathogenic bacteria, viruses, and fungi. As a polyphenolic antioxidant, it demonstrates biological activities, including anticancer and antiaging effects. Resveratrol has better antifungal potency than antibacterial activity, and its MIC values against fungal species (C. albicans, S. cerevisiae, and *Trichosporon beigelii*) range from 20 to >300 μg/mL (60, 61). However, the mechanism by which resveratrol alone has significant antifungal activity against C. albicans remains unclear (62–64). Resveratrol exerts synergistic anticandidal effects when combined with azoles (fluconazole, ketoconazole, and itraconazole) against clinical C. albicans isolates and fluconazole-resistant strains, thereby decreasing the MIC values to 1/4, 1/8, and 1/64, respectively (62). A novel liquid crystal system has been developed to improve the antifungal effects of resveratrol by increasing its contact time with infected buccal sites (63). The *in vitro* mechanism study indicated that resveratrol is a fungal apoptosis inducer and acts through a caspase-dependent pathway. Resveratrol results in the production of ROS and induces the loss of MMP (65). Although clinical trials have proved the safety of resveratrol in the human body, further *in vivo* research on its use for the treatment of fungal infections is still needed (66).

Chiloscyphenol A (CA) is a small-molecule natural product isolated from Chinese liverworts (67). It is effective against *Candida* species, with a MIC range of 8 to 32 μg/mL; an *in vivo* study verified the antifungal effects of CA in a Caenorhabditis elegans model (67). CA is an inhibitor of mitochondrial function by inducing MMP hyperpolarization, increasing ATP and intracellular ROS production, and aggregating Tom70 distribution (67). CA can also destroy the fungal cell membrane. Likewise, floricolin C, a *p*-terphenyl derivative isolated from the fungus *Floricola striata*, exhibits antifungal activity against C. albicans, with an MIC_80_ value of 8 μg/mL, and it induces mitochondrial dysfunction by causing ROS accumulation, which further contributes to cell nuclear dispersion and death (68, 69). Kalopanaxsaponin A (KPA) is a triterpenoid saponin isolated from the stem bark of *Kalopanax pictus*. It shows anticandidal activity, with an MIC range of 8 to 16 μg/mL and prolongs the survival time of C. elegans infected with C. albicans (70). KPA has shown prominent effects against C. albicans pathogenicity by inhibiting adhesion, biofilm formation, and yeast-hyphal transition. KPA also induces ROS accumulation and mitochondrial disturbance, which indicates that mitochondria may be a potential target of KPA. The accumulated ROS could further induce damage to membrane permeability. In addition to the ROS pathway, KPA can directly destroy the C. albicans membrane, causing intracellular trehalose to leak out and a decrease in membrane ergosterol contents (71). Mefloquine (MEF) derivatives, which are antimalarial agents, are acquired through MEF modification and have shown broad-spectrum antifungal activity against a variety of fungi, including some drug-resistant isolates. The combination of MEF derivatives remarkably improved the anti-C. neoformans effects of fluconazole. Furthermore, MEF derivatives have been substantiated to suppress the virulence factors of pathogenic fungi, including reducing the filamentation of C. albicans and the melanization of C. neoformans. These compounds cause mitochondrial dysfunction by disrupting the proton motive force and DNA stability of the mitochondria (72). However, mechanistic studies have revealed the multiple targeting characteristics of MEF derivatives for their effects on both fungal mitochondria and vacuoles.

Papaya (*Carica papaya* Linn.) seed extract (PSE) exhibits antifungal bioactivity against *Candida* species, with an MIC range of 4.0 to 16.0 μg/mL (73). The inhibitory mechanisms of PSE are attributed to mitochondrial dysfunction for ROS generation and MMP collapse (74). Rosmarinic acid (RA) is a polyphenol antioxidant that has been extensively studied for its therapeutic mechanism of action on diseases related to mitochondrial dysfunction (75). RA showed potent anticandidal activity, with an MIC range of 0.1 to 0.2 mg/mL (76). Fialová et al. reported that the quaternary phosphonium salts of RA exhibit stronger antifungal activities than RA (77). The potential anticandidal mechanism of action of RA might be attributed to membrane integrity disruption (not mediated by ergosterol binding) and reduction of mitochondrial activity (76). As the present evidence for mitochondrial damage by RA is limited only to the ≈2-fold reduction of mitochondrial activity, intensive mechanistic studies need to be conducted.

**FIG 1:**
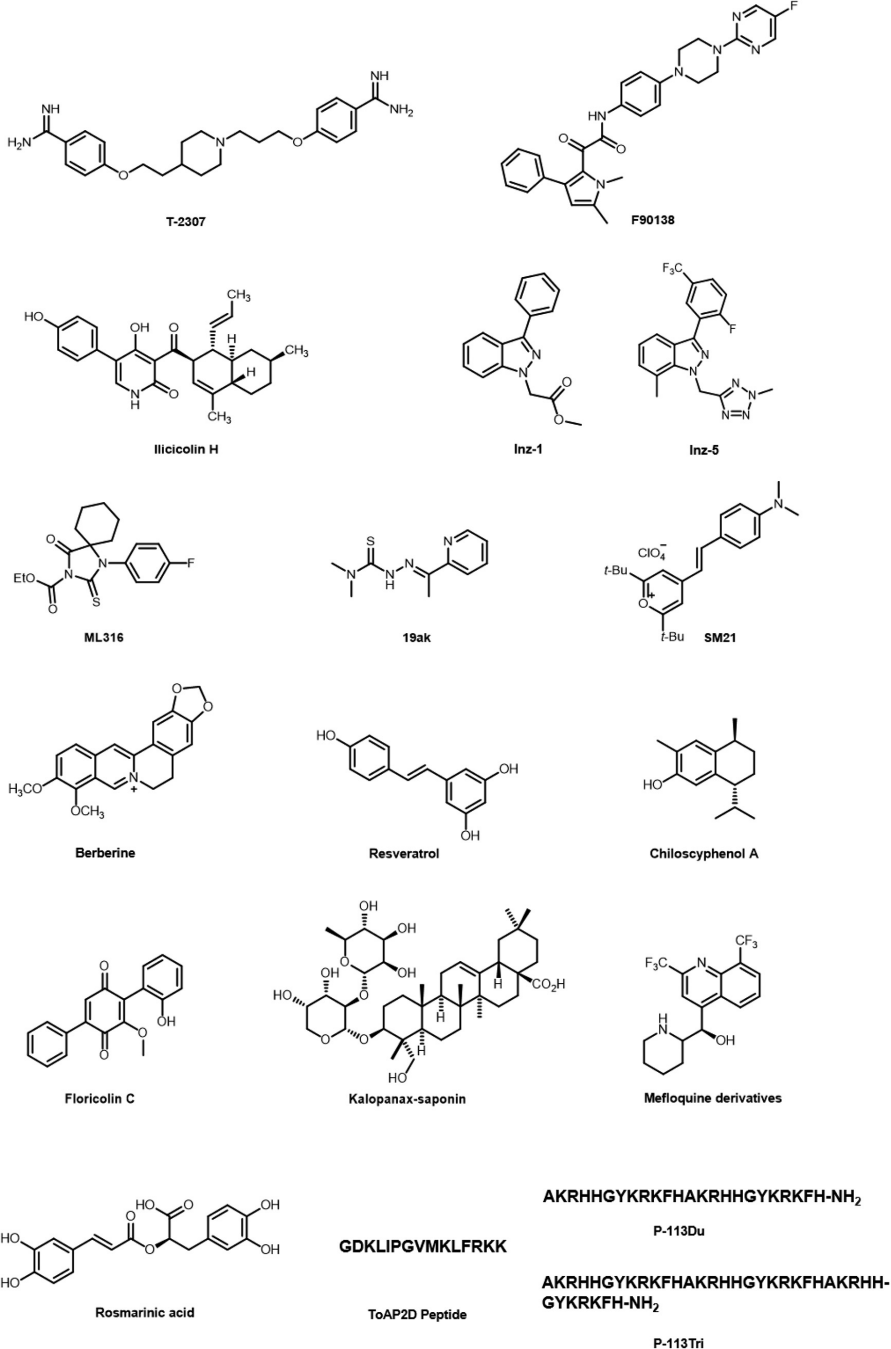
Chemical structures of antifungal agents recently explored that target mitochondria.

Figure 1 provides chemical structures for the above-described candidate compounds and related structures.

## ANTIMICROBIAL PEPTIDES AFFECTING MITOCHONDRIAL FUNCTION

As important components of innate immune responses, antimicrobial peptides (AMPs), such as defensins, cathelicidin, and histatins, reside in most sites of the human body and play a protective role as antifungal agents and immune modulators (78). Histatin 5, a histatin family member, has significant antifungal potency against *Candida* spp., C. neoformans, and A. fumigatus (78, 79). Furthermore, a 12-amino-acid fragment of histatin 5, called P-113, was synthesized and proved to retain strong candidacidal activity without causing adverse effects in clinical trials (80). Studies conducted at a later time reported the production of two derivatives of P113, P-113Du and P-113Tri. Notably, these two newly designed AMPs exhibited superior antifungal activities against planktonic cells, biofilm cells, and clinical isolates of C. albicans as well as non-Candida albicans spp. (80). The latest mechanistic findings uncovered that P-113, P-113Du, and P-113Tri exert antifungal effects by inhibiting the NADH dehydrogenase activity of mitochondrial CI in ETC (11). P-113Du and P-113Tri even blocked a fungus-specific alternative NADH dehydrogenase, which is rarely observed for CI-specific inhibitors (11). Another AMP, named ToAP2D, is also a likely target of fungal mitochondria. It was synthesized based on ToAP2 and exhibited antifungal activity against *Sporothrix globosa* with good serum stability and low toxicity (81). ToAP2D could inhibit the growth of *S. globosa in vitro* and induce its deformation and rupture (82). An *in vivo* study reported that the therapeutic effects of ToAP2D were comparable to those of itraconazole in murine footpad *S. globosa* infection. ToAP2D could trigger the apoptotic pathway in *S. globosa* and induce mitochondrial dysfunction, including a decrease in MMP and ROS accumulation. Moreover, the results of these studies on AMPs have provided further insights into their mitochondria-targeting mechanisms.

**FIG 2:**
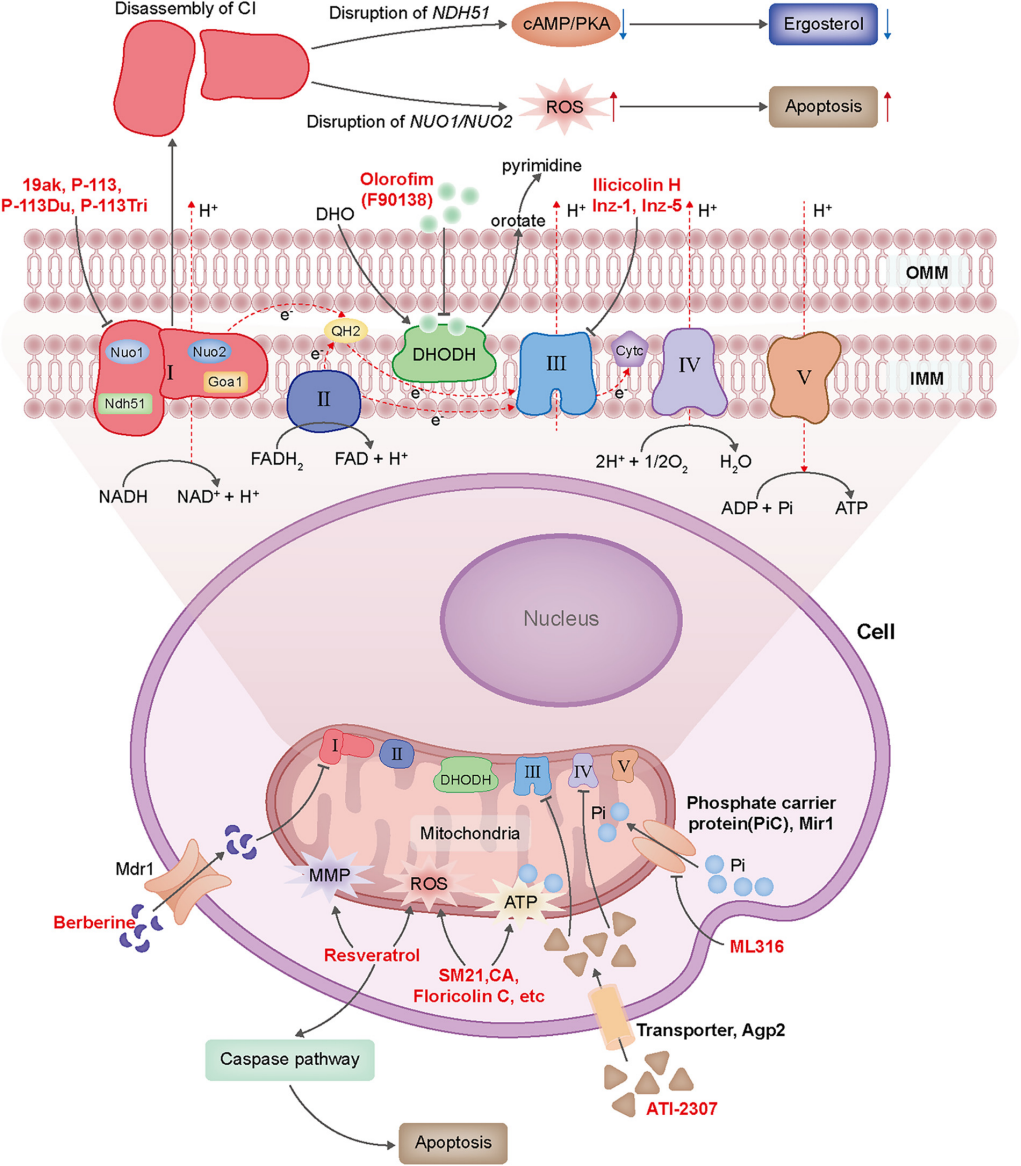
Antifungal agents that target mitochondria and their mechanisms of actions. ATI-2307 acts by inhibiting CIII and CIV of ETC via the transportation of Agp2. F90138 (olorofim) blocks orotate synthesis from DHO by inhibiting the activity of DHODH. Ilicicolin H, Inz-1, and Inz-5 target CIII. ML316 is a novel and selective inhibitor of mitochondrial phosphate carrier protein (PiC) Mir1, which is a shuttle of inorganic phosphate from cytoplasm into mitochondrial matrix for ATP production. CI is also an important target of antifungal agents, for there are some *Candida*-specific proteins (Nuo1, Nuo2, and Goa1) in it. Deficiency of CI subunit proteins will cause CI disassembly and impaired fungal viability. 19ak and antimicrobial peptides P113, P-113Du, and P-113Tri are verified to target CI. Berberine, a natural product, accumulates inside fungal cytoplasm and causes damage of CI. Other compounds, natural products, and peptides, such as SM21, resveratrol, CA, and floricolin C, still lack specific antifungal targets. The antifungal agents are highlighted in red. Abbreviations: I to V, mitochondrial respiration complex I to V; ETC, electron transport chain; DHO, dihydroorotate; DHODH, dihydroorotate dehydrogenase; CA, chiloscyphenol A.

Figure 2 summarized the mechanism of action of these antifungal compounds mentioned above.

### Conclusions and perspectives.

The high prevalence of both fungal infections and antifungal resistance is globally challenging for immunocompromised patients. As recent advances in the development of new antifungal drugs are slow and still confined to producing derivatives of azoles and echinocandins, it is critical to devote efforts to exploring new antifungal drugs, especially for the expansion of the limited antifungal targets at present. More recently, accumulating evidence on mitochondria-mediated pathogenesis has garnered increasing attention in terms of the potential of mitochondria-targeting drugs. Our review provides an overview of the following five categories of ETC proteins: (i) CTG clade-specific mitochondrial subunit proteins (Nue1, Nue2, Nuo3, Nuo4, qec1, Coe1, Coe2); (ii) CTG clade-specific CI regulatory protein (Goa1); (iii) broadly specific proteins in fungi, algae, plants but not in mammals (Nuo1); (iv) fungus-specific proteins (Nuo2), (v) broadly specific proteins, including in mammals (Ndh51) (8, 83). All these mitochondrial proteins in mitochondrial ETC are potential targets for antifungal agents in the future, since their deficiency will result in disastrous damage to cell vitality. However, because of the distinct specificity of these proteins, the antifungal spectrum of agents targeted to them will be different.

In addition, it is noteworthy that mitochondria-targeted antifungal drugs in clinical trials do not act on fungus-specific proteins but are highly selective toward fungal cells (e.g., T-2307 and F90138). Although the antifungal drug candidates ilicicolin H, Inz-1 and Inz-5 act by inhibiting the highly conserved cytochrome *bc*_1_ reductase of CIII, the selectivity of ilicicolin H is approximately 50 times higher than that of Inz-1 or Inz-5. Ilicicolin H is fungus specific, as it does not affect the activity of cytochrome *bc*_1_ reductase in mammalian cells. Inz-1 binds to the Qo pocket of cytochrome *bc*_1_ reductase, and its differential interactions with fungal L275 and human F275 residues may be the key biological event for its fungal selectivity (48). Therefore, the configuration of drug targets and the specific binding sites are crucial elements for the conserved protein to become a potential antifungal drug target and for the drug to exhibit high selectivity. This difference in selectivity limits the clinical application of different drug candidates, owing to high cytotoxicity, even if the targets remain the same.

Although we have provided a detailed list of lead compounds for antifungals, only a small minority of them are being assessed in clinical trials. Thus, it is crucial to make a thorough exploration of possible targets and identify more effective antifungal compounds in the future.
